# Turbidimetric bioassays: A solution to antimicrobial activity detection in asymptomatic bacteriuria isolates against uropathogenic *Escherichia coli*

**DOI:** 10.1002/mbo3.1411

**Published:** 2024-05-06

**Authors:** Ciara Kenneally, Craig P. Murphy, Roy D. Sleator, Eamonn P. Culligan

**Affiliations:** ^1^ Department of Biological Sciences Munster Technological University, Bishopstown Cork Ireland

**Keywords:** antibiotic resistance, antimicrobial peptide, asymptomatic bacteriuria, bacteriocin, uropathogenic *E. coli*, UTI

## Abstract

Traditional bacteriocin screening methods often face limitations due to diffusion‐related challenges in agar matrices, which can prevent the peptides from reaching their target organism. Turbidimetric techniques offer a solution to these issues, eliminating diffusion‐related problems and providing an initial quantification of bacteriocin efficacy in producer organisms. This study involved screening the cell‐free supernatant (CFS) from eight uncharacterized asymptomatic bacteriuria (ABU) isolates and *Escherichia coli* 83972 for antimicrobial activity against clinical uropathogenic *E. coli* (UPEC) strains using turbidimetric growth methods. ABU isolates exhibiting activity against five or more UPEC strains were further characterized (PUTS 37, PUTS 58, PUTS 59, S‐07‐4, and SK‐106‐1). The inhibition of the CFS by proteinase K suggested that the antimicrobial activity was proteinaceous in nature, potentially bacteriocins. The activity of *E. coli* PUTS 58 and SK‐106‐1 was enhanced in an artificial urine medium, with both inhibiting all eight UPECs. A putative microcin H47 operon was identified in *E. coli* SK‐106‐1, along with a previously identified microcin V and colicin E7 in *E. coli* PUTS 37 and PUTS 58, respectively. These findings indicate that ABU bacteriocin‐producers could serve as viable prophylactics and therapeutics in the face of increasing antibiotic resistance among uropathogens.

## Graphical abstract

This study uses turbidimetric bioassays to detect antimicrobial activity in asymptomatic bacteriuria (ABU) isolates against uropathogenic *E. coli* (UPEC). The cell‐free supernatant (CFS) from eight ABU isolates showed promising results. Notably, *E. coli* PUTS 58 and SK‐106‐1 demonstrated enhanced activity in artificial urine medium. The findings suggest a potential use of ABU bacteriocin‐producers as prophylactics and therapeutics amidst rising antibiotic resistance.

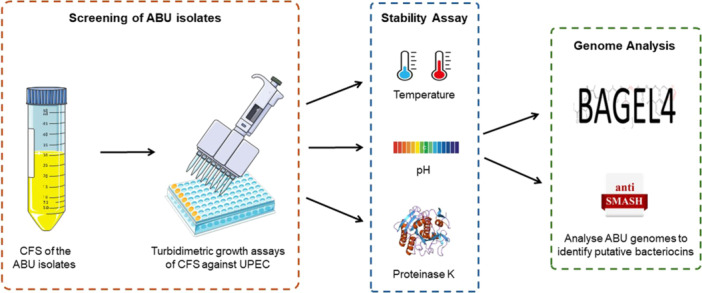

## 1 INTRODUCTION

Uropathogenic *Escherichia coli* (UPEC) is the primary causative agent of urinary tract infections (UTIs) (Hochstedler et al., 2021; Joyce et al., 2022). Approximately 25%–30% of females suffer from recurrent UTIs (rUTIs) 6 months after their initial infection due to the evolution of multi‐drug resistant (MDR) uropathogens (Vagios et al., 2020). Healthcare costs have been significantly impacted. Ireland, France, and the United States have reported their annual primary care costs for UTIs to be €19.2 million, €58 million, and $3.5 billion, respectively (Callan et al., 2014; Flores‐Mireles et al., 2015; François et al., 2016). Given the increasing rates of antibiotic resistance, the search for new and improved alternative therapies must be prioritized.

The human urinary microbiome (urobiome) is home to a diverse range of microorganisms, which represent a significant component of the innate immune response (Jones‐Freeman et al., 2021; Kenneally et al., 2022). An important component of this is bacterial isolates that cause asymptomatic bacteriuria (ABU), which represent a significant component of this innate immunity. ABU occurs when two consecutive urine samples from asymptomatic individuals result in bacterial growth of ≥10^5^ CFU/mL (Nicolle et al., 2005). *E. coli* 83972 was the first strain detected from an individual with ABU and has since been approved as a prophylactic agent for the treatment of lower urinary tract disorders by the European Urology Guidelines (Bonkat et al., 2022). *E. coli* 83972 outcompetes UPEC due to its superior growth rate in urine (Roos et al., 2006), however, the mechanism of action remains unclear. Previous hypotheses include the ability of these strains to outcompete uropathogens for nutrients and attachment sites, or the acquisition of virulence genes (Darouiche & Hull, 2012). Additionally, strains causing ABU may produce antimicrobial peptides (AMPs) known as bacteriocins which kill environmental competitors (Darouiche & Hull, 2012; Stork et al., 2018).

Bacteriocins are ribosomally synthesized peptides produced by bacteria that are toxic to closely related organisms (Dobson et al., 2012). Although current applications of bacteriocins are limited to their use as food preservatives (Benítez‐Chao et al., 2021), they have also exhibited bioactivity against pathogens in murine models. For example, nisin promoted the healing of wounds infected with *Staphylococcus aureus*, pyocins decreased the levels of *Pseudomonas aeruginosa* in lungs, and plantaricin E/F was effective against enteropathogenic *E. coli* (McCaughey et al., 2016; Van Staden et al., 2016). To date, multiple in vivo bacteriocin studies lack data from toxicity and biosafety assays, hindering advancement in clinical trials (Benítez‐Chao et al., 2021). Despite this, approximately 27 AMPs are being tested in clinical trials, with 12 of these at the lead optimization phase (Paulin et al., 2020). It is estimated that all prokaryotes produce at least one bacteriocin (Riley & Wertz, 2002), however, novel bacteriocins are not detected at a rate that represents this. Agar diffusion assays are widely accepted as the most common methods used for the detection of bacteriocins and additional AMPs, however, agar‐based methods are limiting as the complex matrices can interfere with diffusion of AMPs. Efficacy is reduced due to charged sugar and sulfate residues in media that bind to AMPs, preventing them from reaching the target organism (Steinberg & Lehrer, 1997; Twomey et al., 2021). The presence of casein in media causes precipitation of cationic AMPs due to high levels of anionic amino acids (25%) (Mercer et al., 2020; Turner et al., 1998). Additionally, in vitro conditions (temperature, pH, ionic strength, metal ions, and bicarbonate) are not routinely considered when screening for AMPs (Mercer et al., 2020). Discrepancies between these laboratory conditions have been noted when preparing the same agar medium from different brands, interfering with the efficacy of AMPs (Mercer et al., 2020). Whilst agar‐based methods are a highly efficient screening technique, they can underestimate or miss the bioactivity of potential AMPs when such limitations are not considered.

To eliminate the complications associated with agar‐based procedures, broth‐based techniques have been used to quantify the antimicrobial efficacy of crude extracts (Papagianni et al., 2006; Scillato et al., 2021). The turbidimetric method was first used with colicins and has since facilitated the detection of increased antibacterial activity in several studies (Papagianni et al., 2006; Reeves, 1965; Scillato et al., 2021). Turbidimetric assays can be variable when interpreting AMP production, typically resulting from different methods of extracting a crude or pure AMP and differences in the experimental conditions (Papagianni et al., 2006). However, the limitations of the turbidimetric assay are significantly less than the agar diffusion assay, and as such could offer a more reliable detection method for novel bacteriocins.

Previously, we screened ABU isolates for antimicrobial activity against UPEC using traditional agar‐based methods (Kenneally et al, submitted). This screen was conducted to determine the antimicrobial activity of ABU isolates against a bank of MDR UPEC using bacteriocins. Some inconsistencies were observed, as activity detected in the deferred antagonism assay was not reproducible in the well‐diffusion assay. Agar assays identified only four out of nine ABU isolates as antimicrobial producers, whereas bioactivity levels increased in the turbidimetric screen with all nine ABU isolates exhibiting activity against at least two UPECs. Furthermore, bioactivity increased when the CFS was analyzed in artificial urine as two ABU isolates, *E. coli* PUTS 58 and *E. coli* SK‐106‐1 decreased the growth parameters of all eight UPEC strains. *E. coli* SK‐106‐1 displayed bioactivity against five UPEC strains in turbidimetric assays but lacked bioactivity in agar in the previous study. Further analysis was performed using BAGEL4 and antiSMASH 7.0 which identified a microcin operon in *E. coli* SK‐106‐1 similar to the microcin H47 operon in *E. coli* Nissle 1917.

## 2 MATERIALS AND METHODS

### 2.1 Bacterial strains and culture conditions

*E. coli* ABU isolates and UPEC strains used in this study are listed in Appendix Table A1. UPEC indicators previously isolated from patients suffering from recurrent UTI (rUTI) (cystitis) were obtained from the Munster Technological University (MTU, Cork) Culture Collection (Whelan et al., 2020). The ABU isolates were obtained from Forsyth et al. (2018) and Leihof et al. (2021). All *E. coli* strains were cultured in Lysogeny Broth (LB; Neogen) or LB agar (Neogen) at 37°C for 118–24 h with continuous shaking.

Artificial urine was made as previously described by Nzakizwanayo et al. (2019), with alterations (Table 1). All components were obtained from Sigma Aldrich unless otherwise stated. Two separate solutions were prepared and mixed to make the urine medium. Initially, the compounds contained in solution 1 were added to 800 mL of deionized water and the pH was adjusted to pH 5.7 using either sterile 2 M NaOH or 2 M HCl. Subsequently, the final volume was increased to 1 L with deionized water and the solution was sterilized by autoclaving. The components in solution 2 were initially dissolved in 200 mL of sterile deionized water, and the solution was increased to a final volume of 400 mL. Solution 2 was sterilized by filtering through 0.45 µm filters and subsequently, 100 mL of solution 1 was combined with 40 mL of solution 2. Sterile 2 M NaOH or 2 M HCl was used to adjust the pH to 6.1, and 360 mL of sterile deionized water was added to achieve a total volume of 500 mL.

**Table 1:** Composition of the artificial urine medium.

Solution 1		Solution 2	
Potassium dihydrogen phosphate	14.0 g	Calcium chloride	2.45 g
Potassium chloride	8.0 g	Urea	125.0 g
Ammonium chloride	5.0 g	Uric acid	0.15 g
Trisodium citrate	3.25 g		
Sodium sulfate	11.5 g		
Disodium oxalate	0.1 g		
Sodium chloride	23.0 g		
Magnesium chloride	3.25 g		
Creatinine	0.8 g		
Gelatine	25.0 g		
Tryptic soy broth	5.0 g		

### 2.2 In vitro antimicrobial activity of asymptomatic bacteriuria cell‐free supernatants (CFS)

#### 2.2.1 Preparation of ABU CFS

Overnight cultures of ABU isolates in LB were centrifuged at 4000 x *g* for 25 min at 4°C. CFS was retained and adjusted to pH 6.9–7.2 using 1 M NaOH, where necessary. The CFS was filter sterilized using 0.2 µm pore filters. Sterility was confirmed by streaking the CFS on LB agar plates and incubating at 37°C for 16–18 h.

#### 2.2.2 Turbidimetric growth assay in LB

The turbidimetric growth assay was prepared as previously described by Scillato et al. (2021), with modifications. Essentially, UPEC indicator strains were prepared to a population density of 1.5 × 10^8^ CFU/mL/mL in sterile LB. In a 96‐well microtiter plate, 100 µL of each CFS was mixed with 100 µL of each UPEC strain. Growth controls were made by mixing 100 µL of each standardized UPEC strain with 100 µL of LB. To ensure sterility, 200 µL of sterile broth was used as a negative control. Growth was monitored hourly over 24 h at 37°C using a microplate reader (MULTISKAN sky microtiter plate reader, Thermo Scientific; SkanIT Software 6.1) at OD_600nm_. Turbidimetric growth assays were completed in triplicate.

#### 2.2.3 Turbidimetric growth assay in artificial urine

Overnight cultures of the ABU isolates and UPEC strains were prepared in the artificial urine medium and grown in a shaking incubator at 37°C for 16–18 h. CFS was prepared after centrifuging the ABU isolates at 4000 x *g* at 4°C for 25 min. The CFS was filtered through 0.2 µm filters and adjusted to pH 6.9–7.2 with sterile NaOH. UPEC overnight cultures were standardized to a population density of 1.5 ×10^8^ CFU/mL in artificial urine. In a 96‐well microtiter plate, 100 µL of the ABU CFS was combined with 100 µL of each UPEC strain. Growth controls consisted of 100 µL of UPEC in 100 µL of artificial urine. Sterility was measured by adding 200 µL of the artificial urine medium into three wells in the microtiter plate. Absorbance was measured over 24 h at OD_600nm_ using a microtiter plate reader at 37°C.

### 2.3 Stability of antimicrobial activity

#### 2.3.1 pH and temperature sensitivity

To assess the stability of the antagonistic activity, the CFS of each ABU isolate was exposed to different pH values (2.5, 7.0, and 10.0) and temperatures (4°C, 37°C, and 70°C). Sterile 2 M HCl or 2 M NaOH were used to adjust the pH of 5 mL aliquots of the CFSs; the adjusted CFSs were incubated at room temperature (20°C) for 1 h. Each CFS was re‐neutralized to pH 6.5 after the incubation period. Aliquots of the CFS were exposed to the chosen temperatures for 1 h. Growth curves were measured by the turbidimetric growth assay as previously described. CFS from each ABU isolate was mixed 1:1 v/v (100 µL) with the standardized indicator strains in a sterile 96‐well plate, along with their respective controls. Absorbance was recorded over 24 h using a microtiter plate reader (OD_600nm_) at 37°C. Temperature and pH stability assays were completed in triplicate.

#### 2.3.2 Protease sensitivity

The effect of proteinase K on antimicrobial activity was determined using the turbidimetric growth assay. CFS from each ABU isolate was incubated 1:1 v/v with proteinase K solution (1 mg/mL) at 37°C for 2 h (Oliveira et al., 2017). After incubation, CFSs were mixed 1:1 v/v (100 µL) with the standardized UPEC in a 96‐well plate, as previously described, along with their respective controls. Absorbance was read over a 24 h period at OD_600nm_ using a microtiter plate reader at 37°C. Proteinase K sensitivity assays were completed in triplicate.

### 2.4 Bioinformatic analysis

ABU isolates that met the requirements of inhibiting the growth parameters of five or more UPECs were chosen for further analysis. *E. coli* SK‐106‐1 was the only isolate that was not bioactive in agar yet demonstrated efficacy in broth. BAGEL4 (Van Heel et al., 2018) and antiSMASH 7.0 (Blin et al., 2023) were used to predict bacteriocin operons. Identified areas of interest (AOI) were further visualized using the Artemis genome visualization tool (Carver et al., 2012). Where potential antimicrobial peptides were detected, reference sequences were obtained from UniProt (The UniProt Consortium, 2017). AOIs were analyzed on BLASTP to identify sequence homologs (https://blast.ncbi.nlm.nih.gov/Blast.cgi). AOIs were aligned to their reference sequence using the T‐Coffee Multiple Sequence Alignment Server on JalView (Waterhouse et al., 2009).

### 2.5 Statistical analysis

Statistical analysis was performed using RStudio (v 4.2.2; 2022.12.0) (R Core Team, 2022). Data gathered from turbidimetric growth assays were analyzed using the Growthcurver package; both the carrying capacity (K*)* and area under the curve (AUC) were determined (Sprouffske & Wagner, 2016). Normality of distribution and homogeneity of variance were calculated using the Shapiro‐Wilk test and Levene's test, respectively. Normally distributed parameters were subjected to a one‐way analysis of variance (ANOVA) analysis. The bioactivity of the CFSs was assessed against the growth control using post‐hoc tests. The Scheffe post‐hoc test was performed on parameters with a normal distribution and with equal variance. Parameters with a normal distribution which did not assume equal variance were assessed using the Games‐Howell post hoc test. Parameters that were not normally distributed were subjected to a Kruskal–Wallis analysis, with the Conover‐Iman post‐hoc test applied with a Benjamini‐Hochberg adjustment. Statistical significance was defined as having a *p‐*value of <0.05, and results are expressed as the mean ± the standard deviation.

## 3 RESULTS

### 3.1 In vitro antimicrobial activity of cell‐free supernatants from ABU isolates

#### 3.1.1 Turbidimetric growth assay

**Figure 1:**
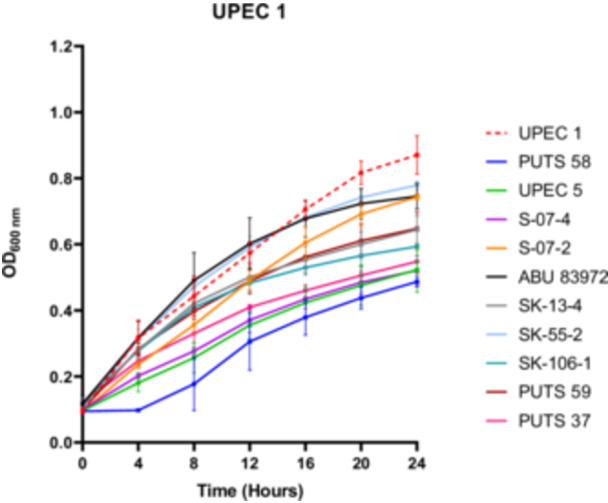
Representative image of the in vitro antimicrobial activity of the cell‐free supernatants from the asymptomatic bacteriuria (ABU) isolates against uropathogenic *Escherichia coli* 1 (UPEC 1). Absorbance was read every hour over 24 h, and every 4 h was plotted for clarity in the interest of including error bars. The red dotted line represents the growth control, UPEC 1 in broth alone. Results are expressed as the mean ± standard deviations of the absorbance values obtained from triplicate experiments.

**Figure A1:**
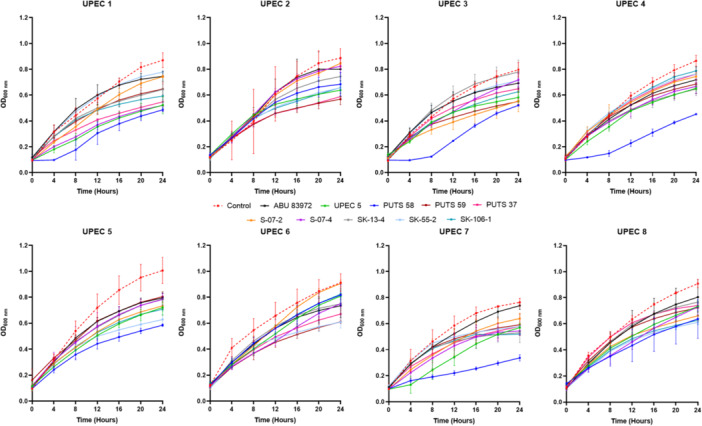
In vitro antimicrobial activity of the cell‐free supernatants from the asymptomatic bacteriuria (ABU) isolates against clinical *Escherichia coli* (UPEC) strains. Absorbance was read every hour over 24 h, and every 4 h was plotted for clarity in the interest of including error bars. The red line represents the growth control, UPEC strains in broth alone. Results are expressed as the mean ±  standard deviations of the absorbance values obtained from triplicate experiments.

A bank of nine ABU isolates from the urogenital tract was screened for antimicrobial activity using their CFS against a panel of eight UPEC indicator strains (Appendix Figure A1). *E. coli* UPEC 5 was also examined for bioactivity as this UPEC strain previously exhibited activity against three of its UPEC counterparts. UPEC 1 was the most susceptible strain, as *E. coli* S‐07‐2 was the only ABU isolate that did not cause a significant growth reduction (*p* < 0.05) (Figure 1). UPEC 3 was the most resistant strain against bacterial interference, as the AUC was the only growth parameter reduced by the CFS from two ABU isolates; *E. coli* PUTS 58 and *E. coli* S‐07‐4. UPEC 6 was also resistant to treatment with ABU CFSs as only three isolates caused significant growth reduction (*p* < 0.05); *E. coli* PUTS 59, *E. coli* PUTS 37, and *E. coli* SK‐55‐2. Every ABU isolate significantly reduced either the K (Table 2) or AUC (Table 3) of at least one UPEC strain. Therefore, further analysis was conducted on the CFSs from ABU isolates that resulted in significant interference with the growth parameters of five or more UPECs. *E. coli* PUTS 58 was the most effective strain at reducing the growth of UPEC, with its CFS inhibiting the growth parameters of six UPEC strains. *E. coli* PUTS 59, PUTS 37, and S‐07‐4 all demonstrated greater levels of antimicrobial activity in the turbidimetric method compared to in agar, reducing the growth parameters of five UPEC strains. *E. coli* S‐07‐2, SK‐13‐4, SK‐55‐2, and 83972 were unable to diffuse in agar, however, these four ABU isolates decreased the growth parameters of UPEC to varying degrees. Notably, *E. coli* SK‐106‐1 displayed antimicrobial activity against five UPEC strains in the turbidimetric assay while in agar‐based methods *E. coli* SK‐106‐1's CFS was unable to diffuse. Therefore, *E. coli* SK‐106‐1 was further analyzed through experimental and bioinformatic approaches to determine the likely source of the antimicrobial activity.

**Table 2:** The effect of the cell‐free supernatants from ABU isolates on the carrying capacity (K) of UPEC strains.

	*K*							
	UPEC 1	UPEC 2	UPEC 3	UPEC 4	UPEC 5	UPEC 6	UPEC 7	UPEC 8
Control	0.796 (0.067)	0.765 (0.056)	–	0.738 (0.032)	0.899 (0.095)	0.777 (0.051)	0.657 (0.026)	0.786 (0.038)
PUTS 58	**0.379** *** (0.019)	**0.548** ** (0.08)	–	**0.394** ** (0.037)	**0.463** *** (0.019)	–	**0.281** *** (0.028)	–
PUTS 59	**0.532** ** (0.054)	**0.433** *** (0.022)	–	–	**0.582** ** (0.016)	**0.488** ** (0.025)	–	**0.569** * (0.019)
PUTS 37	**0.416** *** (0.044)	–	–	–	**0.629** * (0.103)	**0.551** * (0.034)	–	**0.604** * (0.026)
ABU 83972	**0.612** * (0.038)	**0.618** * (0.027)	–	**0.518** * (0.015)	**0.607** * (0.055)	–	–	–
S‐07‐2	–	–	–	–	**0.615** * (0.116)	–	–	–
S‐07‐4	**0.425** *** (0.045)	–	–	–	–		**0.452** * (0.045)	**0.505** ** (0.037)
SK‐13‐4	**0.498** *** (0.018)	**0.519** *** (0.017)	–	–	–	–	**0.431** ** (0.013)	–
SK‐55‐2	–	**0.510** *** (0.079)	–	–	–	**0.472** *** (0.044)	**0.412** ** (0.024)	**0.477** ** (0.026)
SK‐106‐1	**0.452** *** (0.046)	**0.430** *** (0.032)	–	**0.539** * (0.011)	**0.482** *** (0.03)	–	–	**0.489** ** (0.029)
UPEC 5	**0.423** *** (0.045)	**0.476** *** (0.043)	–	**0.535** * (0.032)	–	–	–	**0.601** * 0.036

*Note*: Control indicates the K of each UPEC in broth alone. The asterisks indicate the levels of significance:

*** denotes *p* < 0.001.

** denotes *p* < 0.01.

* denotes *p* < 0.05, and – denotes no statistical significance. (*n* = three biological replicates). Results are expressed as the mean carrying capacity (K) ± standard deviation.

**Table 3:** The effect of the cell‐free supernatants from ABU isolates on the area under the curve (AUC) of UPEC strains.

	UPEC 1	UPEC 2	UPEC 3	UPEC 4	UPEC 5	UPEC 6	UPEC 7	UPEC 8
	AUC	K	AUC	AUC		AUC	AUC	AUC
Control	11.032 (0.756)	–	10.347 (0.584)	10.602 (0.186)	–	12.021 (1.703)	10.438 (1.141)	11.678 (0.600)
PUTS 58	**4.538** *** (0.961)	–	**4.135** *** (0.104)	**3.559** *** (0.246)	–	–	**2.981** * (0.354)	–
PUTS 59	**8.524** * (0.902)	–	–	–	–	**7.529** * (0.714)	–	–
PUTS 37	**6.431** *** (0.04)	–	–	**8.929** ** (1.636)	–	–	–	–
ABU 83972	–	–	–	**8.159** *** (0.598)	–	–	–	–
S‐07‐2	–	–	–	**8.390** ** (0.866)	–	–	–	–
S‐07‐4	**5.907** *** (0.519)	–	**6.428** * (1.149)	**9.498** * (0.537)	–		–	**8.634** * (0.622)
SK‐13‐4	**8.296** * (0.61)	–	–	–	–	–	–	–
SK‐55‐2	–	–	–	–	–	–	–	**8.233** * (0.566)
SK‐106‐1	**7.641** ** (0.189)	–	–	**8.660** ** (0.643)	–	–	–	**7.805** * (0.259)
UPEC 5	**5.676** *** (0.808)	–	–	**7.992** ** (0.277)	–	–	–	**8.000** * (0.309)

Control indicates the AUC of each UPEC in broth alone. The asterisks indicate the levels of significance:

*** denotes *p* < 0.001.

** denotes *p* < 0.01.

* denotes *p* < 0.05, and – denotes no statistical significance. (n = three biological replicates). Results are expressed as the mean area under the curve (AUC) ± standard deviation.

#### 3.1.2 Antimicrobial activity of the cell‐free supernatants in artificial urine

**Figure 2:**
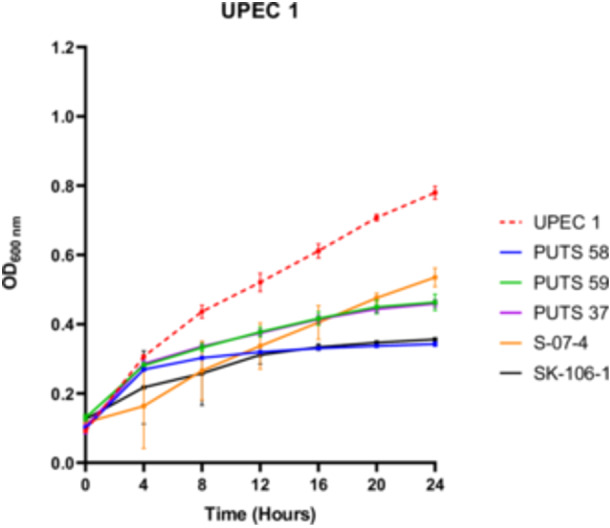
In vitro antimicrobial activity of the cell‐free supernatants against uropathogenic *Escherichia coli* 1 (UPEC 1) in artificial urine. The red dotted line represents the UPEC 1 growth control. Results are expressed as the mean ± standard deviations of the absorbance values obtained from triplicate experiments.

**Figure A2:**
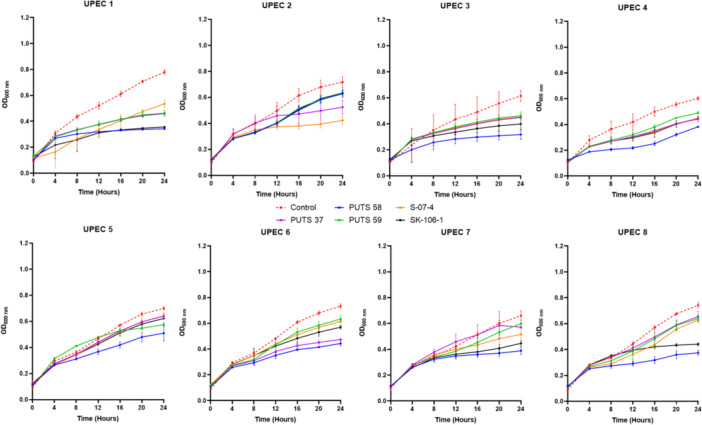
In vitro antimicrobial activity of the cell‐free supernatants against clinical *Escherichia coli* (UPEC) strains in artificial urine. The red dotted line represents the UPEC growth control. Results are expressed as the mean ± standard deviations of the absorbance values obtained from triplicate experiments.

To test the bioactivity of the CFS in a more physiological representative medium, *E. coli* PUTS 58, PUTS 59, PUTS 37, S‐07‐4, and SK‐106‐1 were assessed for their ability to inhibit UPEC growth in artificial urine (Figure 2). The inhibitory potential of the ABU CFSs increased overall in the artificial urine as *E. coli* PUTS 58 and SK‐106‐1 reduced at least one growth parameter of all eight UPEC (Appendix Figure A2). *E. coli* S‐07‐4 was also more bioactive in the artificial urine medium reducing at least one growth parameter of six UPEC (Table 4). The K of UPEC 3 was unaffected in LB, however the CFS of the five ABU isolates significantly reduced the K in urine (*p* < 0.001). Whilst UPEC 6 was resistant to bioactivity in LB, the K of UPEC 6 was reduced by all five ABU isolates and *E. coli* PUTS 59 was the only CFS that did not reduce the AUC (Table 4). UPEC 5 was most resistant to the CFS in artificial urine as only PUTS 59 reduced the K, while PUTS 58 and SK‐106‐1 reduced the AUC. In contrast, UPEC 5 was more sensitive in LB as only S‐07‐4 did not display activity against the UPEC strain.

**Table 4:** In vitro antimicrobial activity of ABU cell‐free supernatants against UPEC in an artificial urine medium.

	UPEC 1		UPEC 2		UPEC 3		UPEC 4		UPEC 5		UPEC 6		UPEC 7		UPEC 8	
	K	AUC	K	AUC	K	AUC	K	AUC	K	AUC	K	AUC	K	AUC	K	AUC
Control	0.688	9.909	0.639	9.303	0.516	–	–	7.459	0.639	8.669	0.675	9.205	0.631	7.778	0.727	8.776
	(0.036)	(0.288)	(0.019)	(0.929)	(0.020)			(0.726)	(0.033)	(0.408)	(0.034)	(0.188)	(0.144)	(0.268)	(0.013)	(0.264)
PUTS 58	**0.226** ***	**4.754** **	**0.567** *	–	**0.193** ***	–	–	**2.998** ***	–	**5.899** ***	**0.324** ***	**5.300** ***	**0.256** **	**5.022** **	**0.256** ***	**4.376** ***
	(0.008)	(0.162)	(0.030)		(0.017)			(0.165)		(0.352)	(0.033)	(0.076)	(0.027)	(0.288)	(0.025)	(0.505)
PUTS 59	**0.324** ***	**5.589** *	**0.563** *	–	**0.339** ***	–	–		**0.446** *	–	**0.540** **	–	–	–	–	–
	(0.024)	(0.289)	(0.047)		(0.040)				(0.018)		(0.041)					
PUTS 37	**0.337** ***	–	**0.380** ***	–	**0.318** ***	–	–	**4.778** ***	–	–	**0.355** ***	**5.760** ***	–	–	–	–
	(0.027)		(0.066)		(0.017)			(0.025)			(0.015)	(0.458)				
S‐07‐4	**0.436** **	**5.505** *	**0.269** ***	**5.362** **	**0.327** ***	–	–	**4.803** ***	–	–	**0.496** ***	**7.206** *	–	–	–	**6.537** *
	(0.046)	(1.216)	(0.027)	(0.414)	(0.039)			(0.417)			(0.007)	(0.255)				(0.546)
SK‐106‐1	**0.224** ***	**4.123** ***	**0.575** *	–	**0.265** ***	–	–	**4.803** **	–	**7.235** *	**0.453** ***	**6.898** *	**0.300** **	–	**0.319** ***	**6.096** **
	(0.021)	(0.579)	(0.017)		(0.042)			(0.020)		(0.204)	(0.018)	(0.882)	(0.022)		(0.011)	(0.210)

The asterisks indicate the levels of significance:

*** denotes *p* < 0.001.

** denotes *p* < 0.01.

* denotes *p* < 0.05, and – denotes no statistical significance. (*n* = three biological replicates). Results are expressed as the mean ± standard deviation.

### 3.2 Stability of antimicrobial activity

#### 3.2.1 pH stability

**Figure 3:**
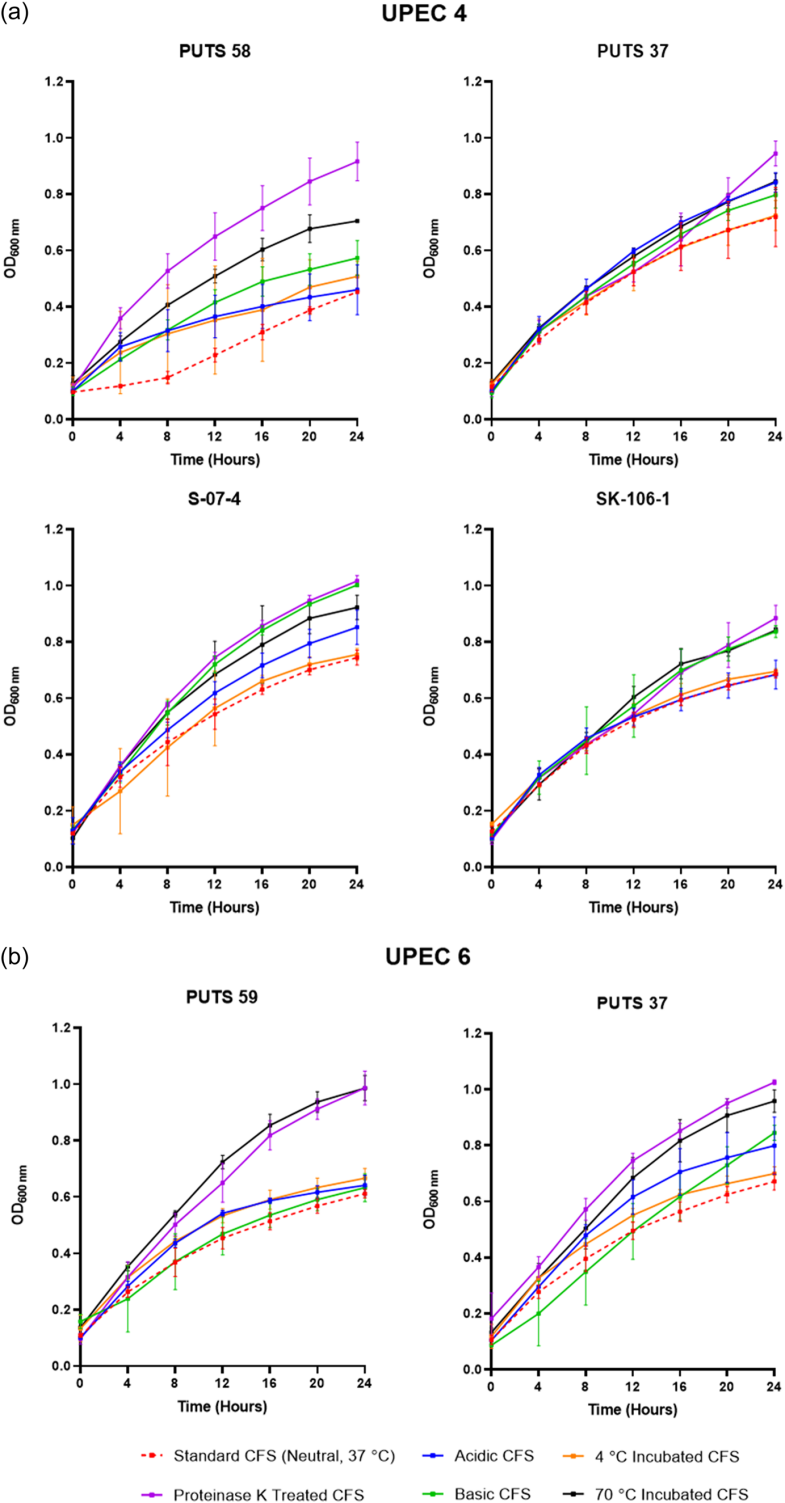
The effect of pH, temperature, and proteinase K on the antimicrobial activity caused by the cell‐free supernatants against uropathogenic *Escherichia coli* (a) UPEC 4 and (b) UPEC 6. The orange dotted line represents the untreated asymptomatic bacteriuria (ABU) cell‐free supernatant. Results are expressed as the mean ± standard deviations of the absorbance values obtained from triplicate experiments.

**Figure A3:**
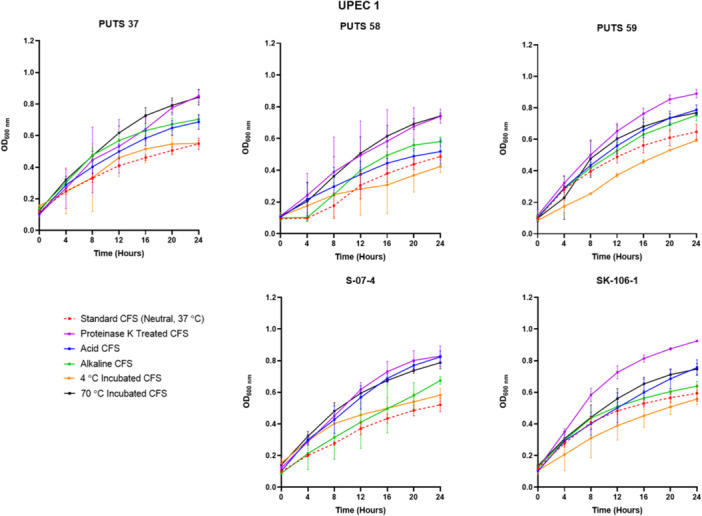
The effect of pH, temperature, and proteinase K on the antimicrobial activity caused by the cell‐free supernatants against *Escherichia coli* 1 (UPEC 1). The red line represents the untreated asymptomatic bacteriuria (ABU) cell‐free supernatant. Results are expressed as the mean ± standard deviations of the absorbance values obtained from triplicate experiments.

**Figure A4:**
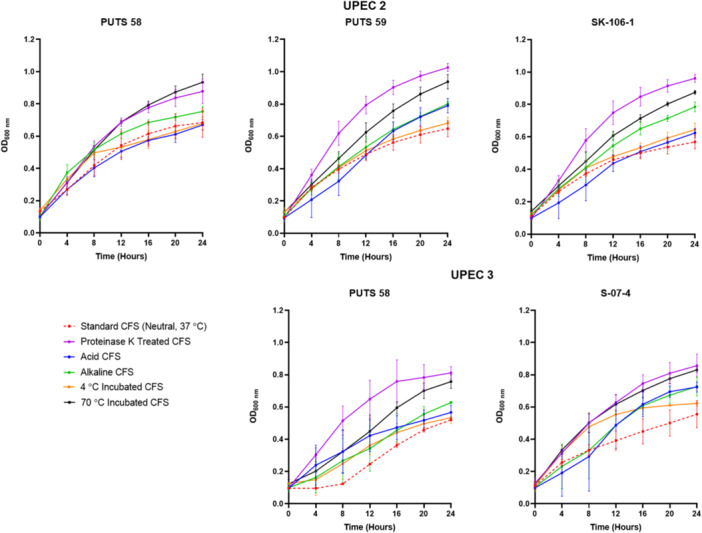
The effect of pH, temperature, and proteinase K on the antimicrobial activity caused by the cell‐free supernatants against *Escherichia coli* 2 (UPEC 2) and 3. The red line represents the untreated asymptomatic bacteriuria (ABU) cell‐free supernatant. Results are expressed as the mean ± standard deviations of the absorbance values obtained from triplicate experiments.

**Figure A5:**
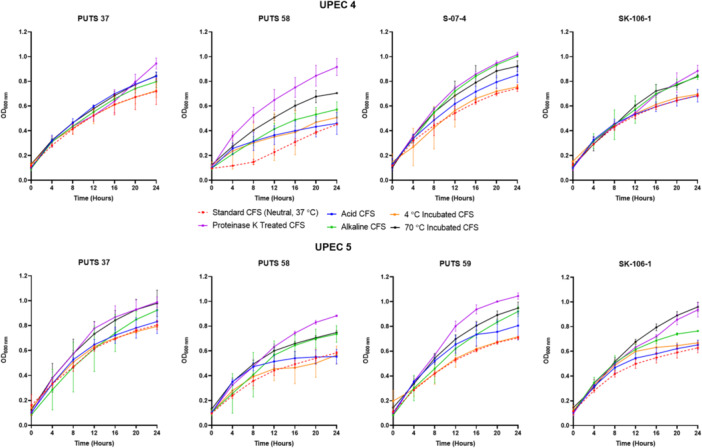
The effect of pH, temperature, and proteinase K on the antimicrobial activity caused by the cell‐free supernatants against *Escherichia coli* 4 (UPEC 4) and 5. The red line represents the untreated asymptomatic bacteriuria (ABU) cell‐free supernatant. Results are expressed as the mean ± standard deviations of the absorbance values obtained from triplicate experiments.

**Figure A6:**
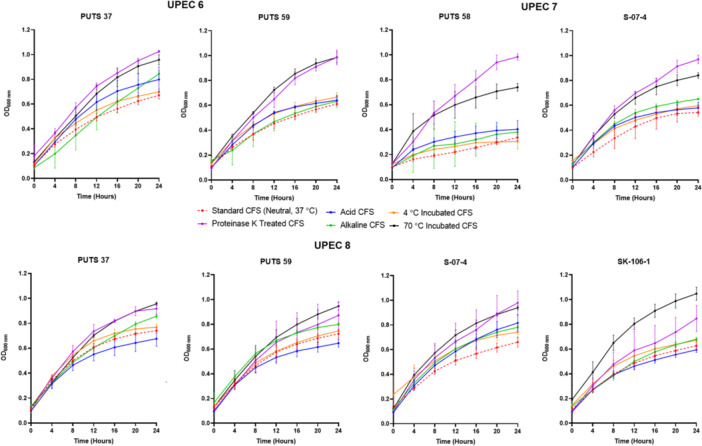
The effect of pH, temperature, and proteinase *K* on the antimicrobial activity caused by the cell‐free supernatants against *Escherichia coli* 6 (UPEC 6), 7 and 8. The red line represents the untreated asymptomatic bacteriuria (ABU) cell‐free supernatant. Results are expressed as the mean ± standard deviations of the absorbance values obtained from triplicate experiments.

The stability of the bioactivity was analyzed by altering the pH and temperature conditions. In general, the CFSs of the ABU isolates did not lose bioactivity when exposed to highly acidic conditions (pH 2.5) (Appendix Figures A3, A4, A5, A6). Bioactivity was lost for the CFSs from *E. coli* PUTS 59 and *E. coli* S‐07‐4 against UPEC 1, and from *E. coli* PUTS 58 and *E. coli* S‐07‐4 against UPEC 3. In contrast, after the CFSs were exposed to a highly basic pH for 1 h (pH 10), UPEC growth reduction by the CFSs was substantially impacted. UPEC 4 and UPEC 6 were the only pathogenic strains whose growth was still reduced by the CFS from every ABU isolate that initially displayed significant interference (Figure 3). Additionally, significant growth reduction (*p* < 0.05) was detected from the CFSs of PUTS 58 and SK‐106‐1 against UPEC 5, SK‐106‐1 against UPEC 8, and the AUC of UPEC 7 was decreased by the CFS of S‐07‐4 (Table 5).

**Table 5:** The effect of pH on the antimicrobial activity of the cell‐free supernatants against UPEC strains.

	UPEC 1		UPEC 2		UPEC 3		UPEC 4		UPEC 5		UPEC 6		UPEC 7		UPEC 8	
	K	AUC	K	AUC	K	AUC	K	AUC	K	AUC	K	AUC	K	AUC	K	AUC
*4°C*																
PUTS 58	**0.151** ***	**6.128** *	**0.544** ***	**0.151** ***	–	–	**0.350** ***	**5.889** ***	**0.437** ***	**8.654** ***	–	–	**0.297** ***	**5.389** ***	**0.479** ***	**8.109** **
	(0.072)	(1.198)	(0.063)	(0.072)			(0.073)	(1.662)	(0.062)	(0.898)			(0.069)	(0.919)	(0.021)	(0.617)
PUTS 59	–	–	**0.612** *	–	–	–	–	–	**0.673** ***	**11.488** ***	**0.520** ***	**8.994** **	–	–	**0.523** ***	**9.248** *
			(0.086)						(0.109)	(1.322)	(0.031)	(0.097)			(0.037)	(0.826)
PUTS 37	**0.576** *	**8.736** *	–	**0.576** *	–	–	–	**9.707** *	**0.692** ***	**11.488** ***	**0.669** *	–	**0.452** *	**8.469** ***	**0.548** ***	–
	(0.046)	(0.937)		(0.046)				(0.780)	(0.094)	(0.963)	(0.098)		(0.065)	(1.083)	(0.065)	
S‐07‐4	–	–	–	–	–	–	**0.623** *	**10.262** *	**0.802** *	**12.512** *	–	–	**0.459** *	**8.408** ***	**0.508** ***	**8.595** **
							(0.035)	(0.485)	(0.039)	(0.201)			(0.052)	(0.631)	(0.043)	(0.746)
SK‐106‐1	–	**9.225** *	**0.715** *	–	–	–	–	**10.685** *	**0.517** ***	**9.105** ***	–	–	–	**9.093** *	**0.445** ***	**7.028** ***
		(1.367)	(0.032)					(1.190)	(0.044)	(0.735)				(0.241)	(0.029)	(0.639)
*70°C*																
PUTS 58	–	–	–	–	–	–	**0.479** ***	**6.819** ***	**0.603** *	–	–	–	–	–	**0.709** *	–
							(0.047)	(0.516)	(0.068)						(0.357)	
PUTS 59	–	–	–	–	–	–	**0.682** *	**10.289** *	–	–	**0.486** ***	**7.448** **	–	–	–	–
							(0.088)	(0.859)			(0.011)	(1.153)				
PUTS 37	–	–	–	–	–	–	**0.718** *	**10.354** *	–	–	**0.765** *	**9.340** **	–	**9.292** *	–	–
							(0.091)	(0.449)			(0.021)	(1.890)		(0.438)		
S‐07‐4	–	–	–	–	–	–	**0.711** *	**9.845** *	–	–	**0.734** *	**10.328** *	–	**8.400** **	–	–
							(0.019)	(0.898)			(0.110)	(2.912)		(0.436)		
SK‐106‐1	–	–	–	–	–	–	**0.777** *	–	**0.626** *	**10.614** *	–	–	–	–	**0.543** **	–
							(0.031)		(0.017)	(0.812)					(0.016)	

The asterisks indicate the levels of significance:

*** denotes *p* < 0.001.

** denotes *p* < 0.01.

* denotes *p* < 0.05, and – denotes no statistical significance. (*n* = three biological replicates). Results are expressed as the mean ± standard deviation.

#### 3.2.2 Temperature stability

The CFSs of the ABU isolates generally remained stable following exposure to temperatures of 4°C (Table 6). Where bioactivity was lost, it often was a case that there was a high standard deviation as the averages were still close to that of other significant values (*p* < 0.05). CFSs had a reduction in efficacy after incubation at temperatures of 70°C (Table 6). *E. coli* PUTS 58 was the only ABU isolate whose CFS was able to withstand exposure to 70°C, exhibiting bioactivity against the AUC of UPEC 3 and the K of UPEC 4.

**Table 6:** The effect of temperature on the antimicrobial activity of the cell‐free supernatants against UPEC strains.

	UPEC 1		UPEC 2		UPEC 3		UPEC 4		UPEC 5		UPEC 6		UPEC 7		UPEC 8	
	K	AUC	K	AUC	K	AUC	K	AUC	*K*	AUC	*K*	AUC	*K*	AUC	*K*	AUC
*4°C*																
PUTS 58	**0.313** ***	–	**0.491** ***	**8.728** *	**0.411** ***	**5.557** ***	**0.432** ***	**5.495** ***	**0.400** **	–	–	–	**0.28** ***	**3.310** ***	**0.521** **	–
	(0.022)		(0.041)	(1.144)	(0.031)	(1.091)	(0.063)	(2.879)	(0.103)				(0.164)	(0.155)	(0.029)	–
PUTS 59	**0.520** ***	**6.510** *	–	–	**0.530** ***	**7.225** **	–	**9.002** **	**0.528** **	**7.705** *	**0.518** ***	**8.773** ***	–	–	–	–
	(0.019)	(0.123)			(0.040)	(1.329)		0.937	(0.097)	(0.533)	(0.052)	(1.169)				
PUTS 37	**0.416** ***	–	**0.512** **	**8.681** **	**0.519** ***	–	**0.518** **	**8.188** ***	–	–	**0.568** **	**9.471** **	–	–	–	–
	(0.018)		(0.045)	(1.272)	(0.018)		(0.025)	(0.526)			(0.061)	(1.200)				
S‐07‐4	**0.419** ***	–	–	–	–	–	**0.575** *	**8.455** ***	**0.568** *	–	**0.644** **	–	**0.455** ***	**7.524** **	**0.62** **	–
	(0.035)						(0.030)	(0.324)	(0.045)		(0.045)		(0.026)	(1.082)	(0.007)	
SK‐106‐1	**0.434** ***	**6.235** *	**0.536** **	**8.287** **	**0.511** ***	**7.906** *	–	–	**0.533** **	–	–	**9.582** **	–	–	**0.502** ***	**8.474** *
	(0.027)	(1.433)	(0.053)	(0.275)	(0.058)	(0.890)			(0.035)			(1.219)			(0.018)	(0.702)
*70°C*																
PUTS 58	–	–	–	–	–	**8.259** *	**0.585** **	–	–	–	–	–	–	–	**0.675** **	–
						(1.262)	(0.031)								(0.042)	–
PUTS 59	–	–	–	–	–	–	–	–	–	–	–	–	–	–	–	–
PUTS 37	–	–	–	–	–	–	–	–	–	–	–	–	–	–	–	–
S‐07‐4	–	–	–	–	–	–	–	–	–	–	–	–	–	–	–	–
SK‐106‐1	–	–	–	–	–	–	–	–	–	–	–	–	–	–	–	–

The asterisks indicate the levels of significance:

*** denotes *p* < 0.001.

** denotes *p* < 0.01.

* denotes *p* < 0.05, and – denotes no statistical significance. (*n* = three biological replicates). Results are expressed as the mean ± standard deviation.

#### 3.2.3 Protease sensitivity

Proteolytic treatment with proteinase K resulted in the loss of inhibitory activity of the CFS from all the ABU isolates against every UPEC (Appendix Figures A3, A4, A5, A6). Interestingly, where a significant result was calculated, the average absorbance readings of the UPEC and ABU CFS combination were larger than the positive control growth of the untreated UPEC in broth.

### 3.3 Bioinformatic analysis

*E. coli* SK‐106‐1 was selected for further analysis since its CFS significantly inhibited the growth parameters of five or more UPEC strains. *E. coli* SK‐106‐1 was chosen for analysis on the basis that it did not demonstrate antimicrobial activity in agar‐based detection assays.

#### 3.3.1 *E. coli* SK‐106‐1

**Figure 4:**
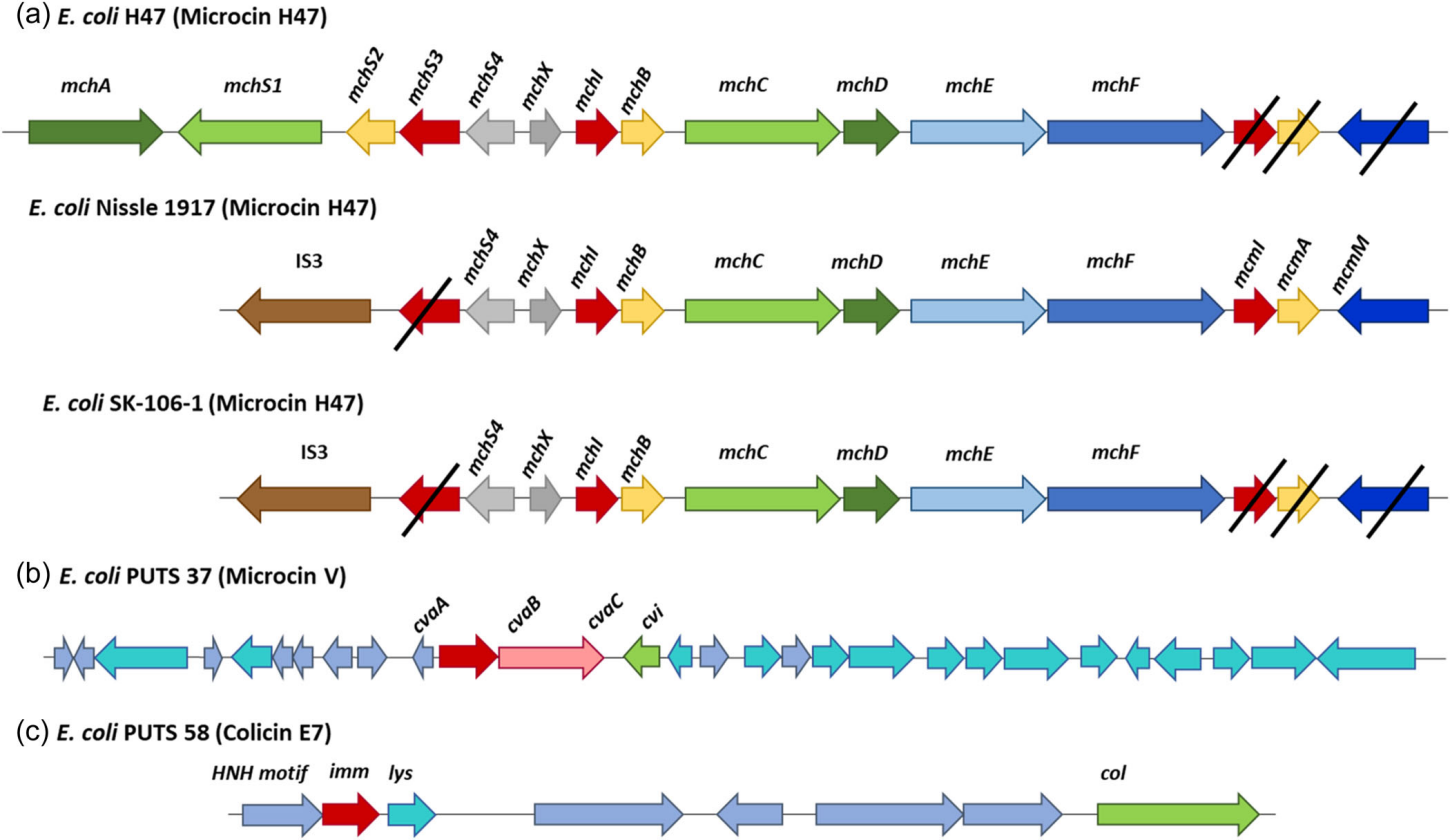
(a) Microcin H47 operon predicted by BAGEL4 and antiSMASH 7.0 in *Escherichia coli* SK‐106‐1. The reference microcin H47 operon and the microcin H47 operon in *E. coli* Nissle 1917 are presented to compare the presence of different genes on the operon. The diagonal black line indicates the absence of that particular gene on the operon in that strain. (b) Microcin V operon predicted in *E. coli* PUTS 37. The genes cvaA and cvaB encode transporter proteins, cvaC encodes the microcin V peptide, and cvi encodes the microcin V immunity protein. (c) Colicin E7 operon predicer in *E. coli* PUTS 58. The col gene encodes the colicin E7 core peptide, imm and lys represent the genes that encode the immunity and lysis proteins, respectively.

BAGEL4 analysis was completed on this genome and seven AOIs were identified. Five out of the seven bacteriocin sequences were missing core peptides, accessory genes, or both. Therefore, it was predicted that these were inactive and/or truncated operons. BAGEL4 predicted a colicin E9 (ColE9) operon, which also encoded colicin immunity proteins. BLASTP analysis of the putative core peptide revealed it was 99.83% identical to a type VI secretion system tube protein TssD, with only one amino acid difference. The predicted immunity proteins showed 100% identity to colicin immunity proteins. A microcin H47 (MccH47) was also predicted by BAGEL4. The predicted core peptide (MchB*)* was 100% identical to an MchB amino acid sequence encoded by the UPEC strains *E. coli* CFT073 (Welch et al., 2002). A colicin E1 (ColE1) core peptide was also predicted upstream on the same operon, however this was 100% identical to a carcinoembryonic antigen (CEA) when analyzed using BLASTP. A MccH47 immunity protein (MchI) was present along with an MchX protein typically identified on MccH47 operons; both were 100% identical to their respective reference proteins. This particular microcin operon also had an IS3 insertion sequence present, which corresponds to a MccH47 operon found in the probiotic *E. coli* strain, Nissle 1917 (Figure 4) (Massip & Oswald, 2020). Additionally, proteins necessary for posttranslational modification (MchC and MchD), and secretion (MchE and MchF) were not identified by BAGEL4 but were manually confirmed on Artemis (Azpiroz et al., 2011) and through BLASTP analysis. Two proteins typically required for biosynthesis during posttranslational modification, MchA and MchS, were not encoded on this operon, as is the case with *E. coli* Nissle 1917 (Vassiliadis et al., 2010).

## 4 DISCUSSION

The urobiome as a source of microbiome‐based therapies has lately become an area of significant interest (Kenneally et al., 2022). Promising urobiome‐based therapies have been discovered from bacteria inhabiting the urogenital tract of asymptomatic individuals that establish bacterial interference against uropathogens (Darouiche et al., 2010; Stork et al., 2018). The success of *E. coli* 83972 as an alternative therapeutic agent prompts investigation as to whether additional ABU isolates demonstrate similar antibacterial effects. This study aimed to characterize the antimicrobial activity of the CFS of uncharacterized ABU *E. coli* strains against UPEC in broth.

All eight ABU isolates reduced the growth parameters of two or more UPEC whilst, in a previous study, the CFS of only four isolates diffused and inhibited UPEC growth using agar‐based techniques (Kenneally et al., submitted). ABU isolates that exhibited activity against five or more UPEC strains were chosen for further analysis, with a particular focus on those showing activity against UPEC 3 and UPEC 6 as they were both resistant to treatment from most ABU isolates. Four strains corresponded to the lead inhibitors in the previous study: *E. coli* PUTS 37, PUTS 58, PUTS 59, and S‐07‐4. *E. coli* SK‐106‐1's CFS was also effective against five UPEC strains in broth. SK‐106‐1's antibacterial efficacy was not previously detected in agar‐based assays; therefore, SK‐106‐1 was chosen for further analysis.

After exposure to acidic conditions (pH 2.5), both *E. coli* PUTS 58 and S‐07‐4 lost their bioactivity against UPEC 3, and *E. coli* S‐07‐4 and PUTS 59 lost their efficacy against UPEC 1. Significance was maintained with every other ABU isolate CFS that was originally detected (*p* < 0.05). Therefore, the active component(s) could be suitable for incorporation into an orally ingested treatment. Bioactivity was mostly weaker in a highly alkaline environment as, after treatment at pH 10, a high proportion (60%) of the CFSs lost activity against UPEC strains they were originally active against. *E. coli* bacteriocins remain stable between pH 2‐9 and bioactivity decreases once a highly‐basic pH (≥10) is encountered (Fathizadeh et al., 2020; Yu et al., 2022). Thus, detergent‐like pH's tend to lower the efficacy of CFSs. Human urine ranges from pH 4.5–8.0, therefore the working range of the bioactive component is similar would suit the physiological pH range (Brooks & Keevil, 1997; Worcester et al., 2018). The tolerability of *E. coli* bacteriocins from highly acidic to weak alkaline conditions means that these bacteriocins are more stable than naturally produced nisin variants that precipitate at physiological pHs (Flynn et al., 2022; Hacker et al., 2015). Previous studies have suggested that the N‐terminal leader protects these bacteriocins against degradation, as the bacteriocin molecule remains inactive until secretion after the N‐terminal leader sequence reaches a specific cleavage site (Duquesne et al., 2007; Rebuffat, 2012).

Bioactivity of the CFSs generally remained significant after exposure temperatures of 4°C, which is ideal for short‐term storage. Typically, *E. coli* bacteriocins are characterized as either heat‐labile colicins or heat‐resistant microcins (Lagos, 2013). The only CFS able to withstand the detrimental effects of heat treatment at 70°C was *E. coli* PUTS 58 when the CFS reduced the growth of UPEC 4 and UPEC 5. *E. coli* PUTS 58's tolerance to heat was not expected, as it is predicted to encode a colicin E7. Antagonistic effects induced by every CFS were lost after proteolytic treatment with proteinase K. Colicins are highly sensitive to proteases whilst microcins are resistant to multiple proteases, only exhibiting susceptibility to proteinase K (Corsini et al., 2010; Hahn‐Löbmann et al., 2019). Although microcins are assumed to be more stable than colicins in relation to pH, temperature, and protease stability, such assumptions have yet to be rigorously validated (Parker & Davies, 2022). For instance, microcins N and L have both exhibited susceptibility to proteases that microcins are typically resistant to (Corsini et al., 2010; Pons et al., 2004). Overall, this further supports our conjecture that the ABU isolates are producing antimicrobial peptides which denature when encountering proteinase K and are generally heat labile. Additionally, novel colicins and microcins exhibiting different stability characteristics are likely to exist and are yet to be discovered.

Mercer and co‐authors (2020) previously stated that for antimicrobial screening methods to be more reliable, media conditions should resemble physiologically relevant environments. Thus, the bioactivity of the CFSs against UPEC was further analyzed in an artificial urine medium. *E. coli* PUTS 58 and *E. coli* SK‐106‐1's antimicrobial activity increased in the artificial urine medium as they both inhibited at least one growth parameter of all eight UPECs. Furthermore, antimicrobial activity was demonstrated against the UPEC strains that were more resistant in LB: UPEC 3 and UPEC 6. Bacteriocin activity has not been evaluated in artificial urine, however, bacteriophages targeting *Pseudomonas aeruginosa* and *Proteus mirabilis* have exhibited activity against biofilm formation in artificial urine (Lehman & Donlan, 2015). Increased activity could be due to a number of factors such as artificial urine being a more suitable matrix for the ABU CFSs to exhibit activity. Artificial urine and human urine contain fewer carbon atoms and amino acids than LB (Sezonov et al., 2007), hence, UPEC grows to a higher density in LB. Additionally, colicins and microcins are routinely produced as a stress response in nutrient‐limiting environments (Marković et al., 2022; Riley, 2009), such as urine. However, it should be noted that the composition of human urine is more variable based on gender, age, ethnicity, food and fluid intake, infection, medication, and exercise (Reitzer and Zimmern, 2020; Taylor & Curhan, 2007; Wang et al., 2021). These factors could interfere with the efficacy of the ABU isolates in human urine.

*E. coli* SK‐106‐1's genome was analyzed using BAGEL4, as out of five isolates that were examined further, SK‐106‐1 did not show any antagonistic activity when used in agar‐based assays. This could be due to interactions of a potential bacteriocin with sugar or sulfate residues, or an unsuitable ionic charge (Bubonja‐Šonje et al., 2020; Mercer et al., 2020; Steinberg & Lehrer, 1997). A lack of activity could also be due to unsuitable media components, as it has been hypothesized that antimicrobial activity increases in a more physiologically representative medium (Mercer et al., 2020).

Two putative bacteriocin core peptides were identified in SK‐106‐1, a ColE9 and a MccH47. BLASTP revealed the ColE9 core peptide was 99.83% identical to a type VI secretion system tube protein TssD (hemolysin‐coregulated protein [Hcp]) as there was only one amino acid difference. Hcp contributes to interbacterial interactions, allowing a donor to deliver virulent proteins and toxic effectors to receiving organisms (Russell et al., 2014). The MccH47 core peptide was 100% identical to its reference protein. However, the MccH47 operon predicted in SK‐106‐1 lacked two proteins required for posttranslational modification, MchA and MchS*. E. coli* Nissle 1917, the active component of the probiotic Mutaflor, also encodes a MccH47 operon lacking both of these genes (Massip & Oswald, 2020). Furthermore, an IS3 insertion sequence was present on the operon upstream of the core peptide which is also syntenic to the operon in *E. coli* Nissle 1917 (Vassiliadis et al., 2010). The proteins McmI, McmA, and McmM were also absent. This truncation distinguishes H47 operons from other catechol‐microcins. MccH47 is categorized as a catechol‐microcin, post‐translationally modified with an enterobactin moiety (Baquero et al., 2019; Vassiliadis et al., 2011). Hence, the acquisition of MccH47 is considered a virulence mechanism in UPEC (Massip & Chagneau, Boury, et al., 2020; Massip & Oswald, 2020), facilitating iron uptake in iron‐limiting environments, such as urine, during pathogenesis. MccH47 is an advantageous bioactive component, disguising itself from target bacteria by exploiting the siderophore moiety to bind to catecholate siderophore receptors (Baquero et al., 2019; Vassiliadis et al., 2011). This allows the microcin to target the ATP synthase F_0_ proton channel, triggering the entry of protons to dissipate the membrane potential (Massip & Oswald, 2020; Rodríguez & Laviña, 2003).

Whilst agar diffusion assays are routinely used for detecting bacteriocin producers, the turbidimetric assay provided a more informative screen. CFSs from all nine ABU isolates were shown to be significant against two or more UPEC as opposed to only four ABU isolates exhibiting bacteriocin‐like diffusion in agar. Notably, the antimicrobial activity caused by *E. coli* SK‐106‐1's CFS was only detected in turbidimetric assays. Turbidimetric screens eliminate the issues encountered with agar diffusion methods and are advantageous as they can quantify the efficacy of bacteriocin producers. This theory is reviewed by Mercer et al. (2020) who suggest that the standard protocols for the detection of AMPs require rigorous review. However, the turbidimetric screen is more sensitive than agar‐diffusion methods (Scillato et al., 2021; Scorzoni et al., 2007) which could give an unrealistic evaluation of the antimicrobial potential. Therefore, a combination of both methods to identify the most bioactive producer strains would be the best approach at present. Ultimately, revising what is deemed a standard protocol will aid the development of more reliable screening methods, and in turn, accelerate the detection of novel biotherapeutics.

Overall, our screen of ABU isolates against UPEC resulted in the identification of putative bacteriocin operon in *E. coli* SK‐106‐1. The presence of bacteriocins likely enhances ABU bioactivity as SK‐106‐1 were more effective than the prototypical ABU isolate, *E. coli* 83972. However, determining the peptide mass to confirm the presence of the putative bacteriocin is warranted as other antimicrobial compounds could be present in the CFS. Nonetheless, *E. coli* SK‐106‐1 and PUTS 58 exhibited the greatest levels of antimicrobial activity in broth and PUTS 58 was also the most bioactive ABU isolate in agar. A colicin E7 (ColE7)‐like operon was previously identified in *E. coli* PUTS 58 (Figure 4), whilst *E. coli* SK‐106‐1 encoded the MccH47. Typically, MccH47 is associated with urovirulence in relation to UPEC (Massip & Chagneau, Boury, et al., 2020; Massip & Oswald, 2020). Although ColE7 is not classed as a virulence trait, the production of another ‘E’ colicin, colicin E1, has been categorized as a potential virulence factor in UPEC strains (Šmajs et al., 2010). Despite this, both ColE7 and MccH47 producers have exhibited preliminary activity against uropathogens (Budič et al., 2011; Storm et al., 2011). As significant reductions (*p* < 0.05) of the growth parameters (K and AUC) were detected, further analysis is warranted with a larger bank of uropathogens to determine if the CFS is bactericidal or bacteriostatic. Subsequently, an expanded virulence profile will determine whether the ABU isolates are a safe biotherapeutic for the treatment of UTIs. Bacteriocin‐producers are not suitable as probiotics if their antimicrobial resistance and virulence repertoire is inappropriate, thus purification is a strategy being employed to develop therapeutic bacteriocins (Cameron et al., 2019). Both nisin and pediocin PA‐1, two FDA‐approved bacteriocins, are permitted for use as partially purified bacteriocin. Partial purification will still separate the antimicrobial component from the host and is a less time‐consuming and more cost‐effective approach (Todorov et al., 2022). Engineering a probiotic or generally recognized as a safe (GRAS) organism to encode the bacteriocin could also increase the antimicrobial potential (Kenneally et al., 2022). A recombinant *Lactobacillus brevis* DT24 expressing colicin E2 has exhibited increased inhibition of UPEC (Trivedi et al., 2014). Employing a GRAS organism as a recombinant bacteriocin producer would permit the use of a safer microbial host as an active biotherapy for treating UTIs.

## AUTHOR CONTRIBUTIONS

**Ciara Kenneally**: Conceptualization; methodology; writing—original draft; data curation; investigation; formal analysis; project administration; funding acquisition. **Craig P. Murphy**: Writing—review and editing; formal analysis; supervision. **Roy D. Sleator**: Writing—review and editing; supervision. **Eamonn P. Culligan**: Supervision; writing—review and editing; funding acquisition.

## CONFLICT OF INTEREST STATEMENT

None declared.

## ETHICS STATEMENT

None required.

## Data Availability

Raw reads and assembled contigs are available under the accession numbers JBAKMN000000000, JBAKMO000000000, JBAKMP000000000, JBAKMQ000000000, DAEBDI000000000, DAEBDQ000000000, DAEBEH000000000, DAEBDH000000000, DAEBEA000000000, NC_017631.

## APPENDIX A

**Table A1:** E. coli ABU and indicator strains used in this study.

ABU isolates	Indicator strains
ABU 83972	UPEC 1
PUTS 37	UPEC 2
PUTS 58	UPEC 3
PUTS 59	UPEC 4
S‐07‐2	UPEC 5
S‐07‐4	UPEC 6
SK‐106‐1	UPEC 7
SK‐55‐2	UPEC 8
SK‐13‐4	
